# Surface-active ionic liquids for palladium-catalysed cross coupling in water: effect of ionic liquid concentration on the catalytically active species†
†Electronic supplementary information (ESI) available. See DOI: 10.1039/c7ra07757b


**DOI:** 10.1039/c7ra07757b

**Published:** 2017-08-22

**Authors:** Meltem Taskin, Alice Cognigni, Ronald Zirbs, Erik Reimhult, Katharina Bica

**Affiliations:** a Institute of Applied Synthetic Chemistry, Vienna University of Technology, Getreidemarkt 9/163, 1060 Vienna, Austria. Email: katharina.schroeder@tuwien.ac.at; Fax: +43 1 58801 16360; Tel: +43 1 58801 163601; b Institute for Biologically Inspired Materials, Department of Nanobiotechnology, University of Natural Resources and Life Sciences, Vienna, Muthgasse 11-II, A-1190 Vienna, Austria

## Abstract

We report the design and synthesis of surface-active ionic liquids for application in palladium-catalyzed cross coupling reactions. A series of dodecylimidazolium-based ionic liquids were applied as additives in the Heck reaction of ethyl acrylate and iodobenzene, and high yields of >90% could be obtained in water without the addition of further ligands. Our results indicate that the ionic liquid concentration in water is the key factor affecting the formation of the catalytically active species and hence the yield. Moreover, imidazolium-based ionic liquids that are able to form a carbene species differ significantly from conventional cationic surfactants, as a concentration dependent formation of the N-heterocyclic carbene complex was observed.

## Abstract 2

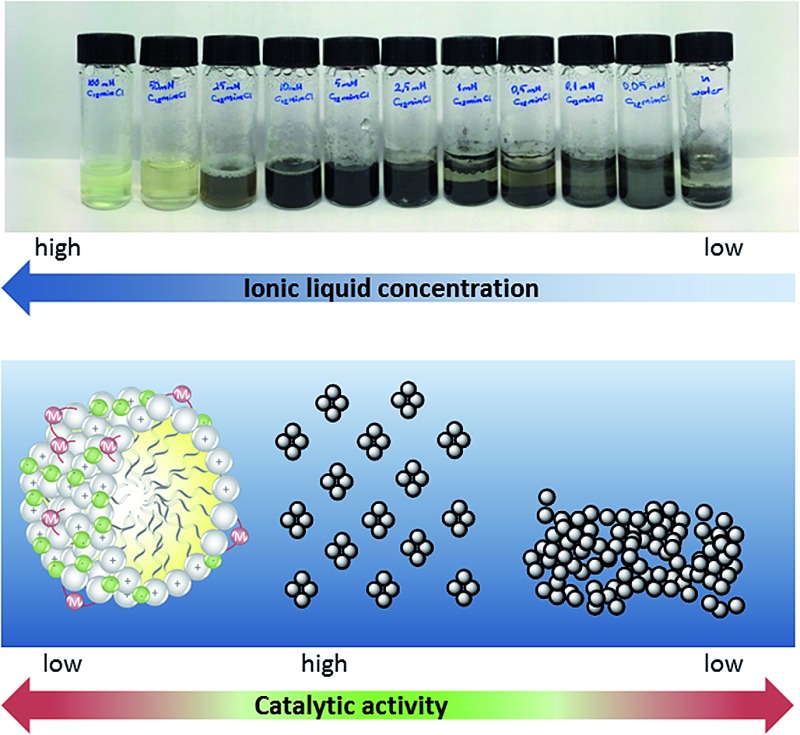
We report the design and synthesis of surface-active ionic liquids for application in palladium-catalyzed cross coupling reactions.

## Introduction

With the recent focus on sustainable chemistry, considerable effort has been spent on the search for novel reaction media, *e.g.* ionic liquids for improved performance, catalyst recycling and product separation. In a seminal paper in 1999, Carmichael *et al.* demonstrated the potential of ionic liquids in multiphase Heck reactions and opened the field for palladium-catalysed cross-coupling in ionic liquids.^1^ A number of benefits in terms of catalyst reuse and facilitated product separation based on the distinctive phase behaviour exist in ionic liquids.^2^ Moreover, changes in reactivity compared to traditional molecular solvents were observed based on the ionic nature of the reaction media.^3^ In fact, it is now well recognized that ionic liquids are far from innocent when used as solvents in transition metal chemistry and can actively participate in catalysis.^4^ This is particularly true for 1,3-dialkylimidazolium-based ionic liquids that are likely to take an active part in a reaction,^5^,^6^ for example *via* the formation of a N-heterocyclic carbene complex with a number of transition metals,^7^,^8^ substrates^9^ or gases such as CO_2_.^10^

In parallel to the developments in the area of ionic liquids, research has focused on the use of water as the most abundant and non-toxic solvent for transition metal catalysis.^11^ Aqueous organic solvents, *e.g.* H_2_O–dimethylformamide, but also aqueous organic biphasic systems in combination with water-soluble ligands were successfully applied in palladium catalysis under aqueous conditions, although the nature of the active catalyst in water is not always completely understood.^12^ The limited solubility of many organic starting materials can be overcome by the addition of surfactants, and ground-breaking contributions by Lipshutz and co-workers revealed the potential of aqueous-micellar systems for palladium-catalysed cross-coupling.^13^ The development of designer surfactants based on nature-derived tocopherol cores resulted in outstanding catalytic activity for a number of transition-metal catalysed processes and a revival of interest in micellar catalysis.^14^

The scope of ionic liquids as alternative solvents has only been recently expanded to include their well-defined mixtures with water.^15^ While the typical properties of ionic liquids continue to exist at low to moderate water content, electrostatic screening is progressively attenuated with higher dilution. For short chain imidazolium-based ionic liquids, the interaction with water is mediated by the anions as they are capable of forming hydrogen bonds with water.^16^ However, these aqueous ionic liquid mixtures seem to resemble polar mixtures rather than behave as normal electrolytes.^17^ Increasing the alkyl chain length of the imidazolium cations leads to aggregation and consequently to the formation of micelles.^18^ This provides a dual beneficial approach for synthesis and catalysis, as surface-active ionic liquids in water cannot only overcome the price or viscosity issues of pure ionic liquids as solvents, but form nanoreactors that can easily be tuned and modified to fit the reaction requirements.^19^

Consequently, the aggregation behaviour of ionic liquids with an alkyl chain longer than eight carbon atoms in water has been increasingly investigated in the past years, particularly for imidazolium based derivatives.^20^–^23^ Micellar aggregates of surface-active imidazolium-based ionic liquids can increase the solubility of organic compounds in water,^24^ which renders them ideally suited for the extraction of natural compounds from biomass.^25^–^28^ Moreover, the high tunability of surface-active ionic liquids provides a powerful tool for synthesis and catalysis in water. A strong impact on activity and selectivity through specific interactions between micelle, substrates and catalysts has been observed by a number of authors.^29^–^34^

In our previous work, we demonstrated that the self-organization of surface-active 1-alkyl-3-methylimidazolium salts [C_*n*_mim]X can considerably increase the rate constants of organic and transition metal catalyzed reactions compared to neat water and can affect the outcome of the reactions.^35^,^36^ Here, we explore their application towards palladium-catalysed cross coupling and present a striking influence of ionic liquid concentration on the catalytic performance.

## Results and discussion

**Fig. 1:**
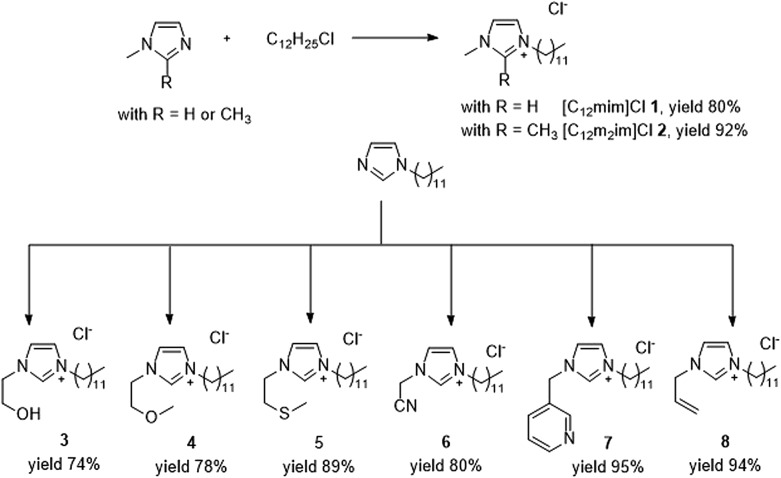
Selection and synthesis of amphiphilic imidazolium chlorides **1–8** for palladium-catalysed cross coupling in aqueous solution. CMC: critical micelle concentration determined *via* surface tension measurements at 25 °C. See ESI Table S1† for more information.

The choice of ionic liquid type can be crucial for the success or failure of any organic reaction. We selected a set of surface-active imidazolium-based ionic liquids **1–8** that are composed of a hydrophilic imidazolium head group and a hydrophobic dodecyl chain (Fig. 1). Consequently, they exhibit amphiphilic behaviour, forming micelles in aqueous solution. Critical micelle concentrations (CMC) typically between 5 and 15 mM have been reported by a number of authors (see ESI Table S1†).^20^,^37^–^40^ We were particularly interested in surface-active ionic liquids with imidazolium cores and optional side-chain functionalization, as they offer the possibility for N-heterocyclic carbene (NHC) formation.^41^,^42^ Carbene formation in imidazolium salts typically occurs by deprotonation of the acidic proton in position 2, although carbenes on position 4 and 5 have been described as well.^10^ However, these so-called normal carbenes are typically less stable and hardly observed in an aqueous environment.^42^ For comparison, we have also included the 1,2-dimethylimidazolium-based ionic liquid [C_12_m_2_im]Cl in our selection.

Unfunctionalized surface-active ionic liquids [C_12_mim]Cl **1** and [C_12_m_2_im]Cl **2** were prepared *via* the direct alkylation of 1-methylimidazole or 1,2-dimethylimidazole with dodecyl chloride. For the functionalized ionic liquids **3–8** we relied on a two-step procedure *via* the pre-formation of *N*-dodecylimidazolium which was then further reacted with the appropriate halides. This gave straight forward access to the desired functionalized surface-active ionic liquids. In any case, the surface-active ionic liquids were obtained as colourless crystals in excellent yields after recrystallization from suitable solvents.

**Fig. 2:**
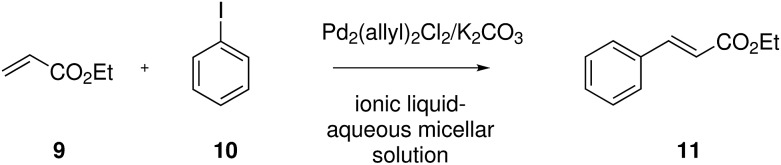
Palladium-catalysed Heck reaction of ethyl acrylate **9** and iodobenzene **10** in ionic liquid-aqueous micellar solutions.

Initial experiments on the Heck reaction were performed in 100 mM solutions of surface-active ionic liquids **1–8** in water, as this concentration is well above the critical micelle concentrations of the respective surface-active ionic liquids (Fig. 2). The reaction of ethyl acrylate and iodobenzene using Pd_2_(allyl)_2_Cl_2_ as the metal source was performed at 80 °C and revealed a surprising behaviour (Table 1). While high yields at 100 mM concentration could be obtained with the ionic liquid [C_12_m_2_im]Cl **2**, all other ionic liquids (**1** and **3–8**) that have a proton available at C-2 failed as reaction media and were outperformed even by pure water. In contrast, a completely different behaviour was observed when lowering the concentration to 10 mM. In the case of the surface-active ionic liquid [C_12_mim]Cl **1** the yield increased to 51%. This increase of yield at a lower surfactant concentration was observed for all side-chain functionalized ionic liquids with the exception of the pyridine derivative **7**, and the best yields were obtained with the nitrile-functionalized imidazolium chloride **6**. In contrast, the yield of ethyl cinnamate **11** was reduced to 25% when lowering the concentration of [C_12_m_2_im]Cl **2** to 10 mM.

**Table 1:** Impact of surface-active ionic liquid concentration in the palladium-catalyzed Heck reaction of ethyl acrylate **9** and iodobenzene **10**

Entry^*a*^	Surfactant	Conc. (mM)	Yield^*b*^ (%)
1	[C_12_mim]Cl **1**	100	<1
2	[C_12_m_2_im]Cl **2**	100	92
3	**3**	100	<1
4	**4**	100	<1
5	**5**	100	<1
6	**6**	100	<1
7	**7**	100	<1
8	**8**	100	<1
9	CTAB	100	52
10	[C_12_mim]Cl **1**	10	51
11	[C_12_m_2_im]Cl **2**	10	25
12	**3**	10	51
13	**4**	10	16
14	**5**	10	12
15	**6**	10	68
16	**7**	10	<1
17	**8**	10	11
18	CTAB	10	20
19	Pure H_2_O	—	3

^*a*^Reactions were carried out with 1 mmol ArI, 1.5 mmol ethyl acrylate and 1.5 mmol K_2_CO_3_ in 5 mL of aqueous ionic liquid solution at 80 °C under air for 180 min.

^*b*^Yield determined *via* HPLC using phenol as internal standard.

**Fig. 3:**
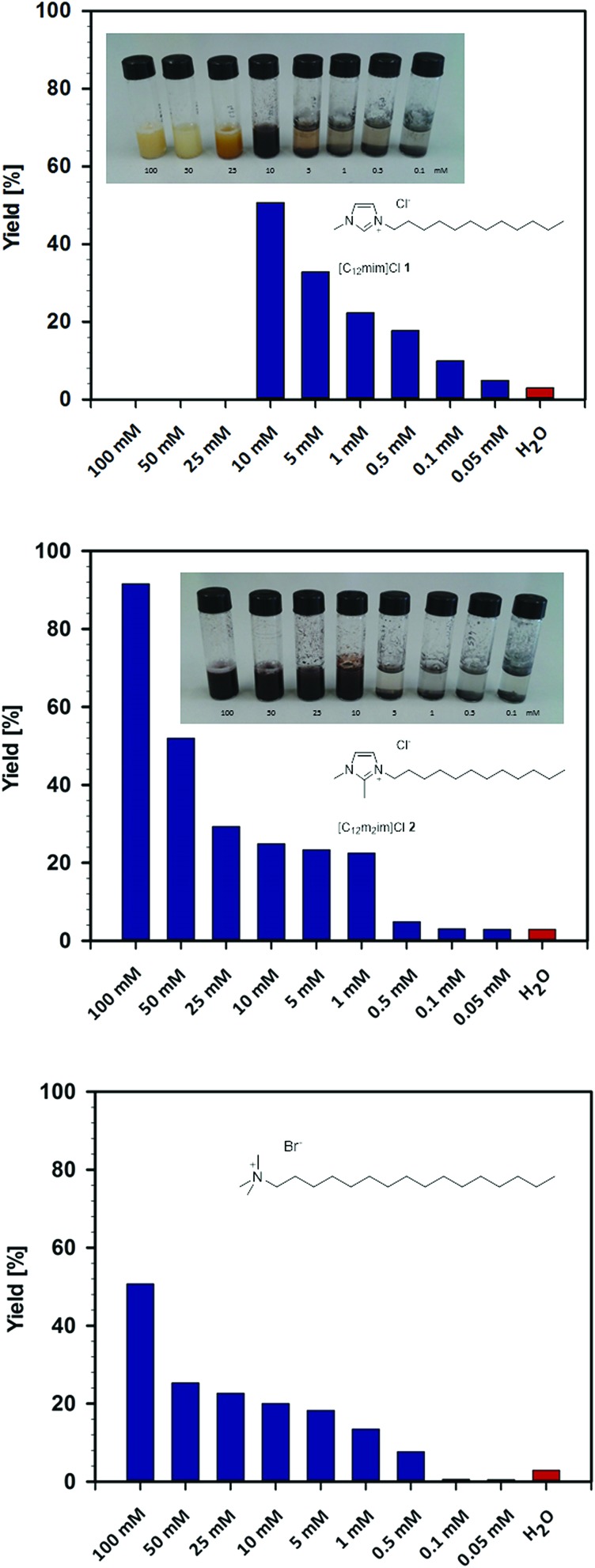
Concentration dependence of the Heck reaction of ethyl acrylate **9** and iodobenzene **10** in aqueous solutions of ionic liquids [C_12_mim]Cl **1** (top) and [C_12_m_2_im]Cl **2** (middle) and the conventional surfactant CTAB (bottom).

Further investigation revealed a striking influence of the concentration of the surface-active ionic liquids and a fundamentally different behaviour between the C-2 protonated and methylated ionic liquids (Fig. 3). In the case of [C_12_mim]Cl **1**, a decrease of the concentration in water resulted in a sudden increase in catalytic activity at 10 mM. A maximum yield of 51% was found at 10 mM, whereas yields gradually dropped with a further decrease of the concentration to eventually reach the values that were obtained with pure water. This rather unique behaviour is entirely different to the concentration effect observed in the C-2 methylated ionic liquid [C_12_m_2_im]Cl **2**, for which high yields of up to 92% were observed that gradually decreased over the entire concentration range, as would be expected in the presence of a commonly used surfactant in water. In fact, when comparing [C_12_m_2_im]Cl **2** with the conventional cationic surfactant cetyltrimethylammonium bromide (CTAB), a similar trend was observed, although the ionic liquid performed significantly better and gave almost twice the yield. This outstanding performance of imidazolium-based surface-active ionic liquids compared to conventional ammonium-based surfactants is in accordance with our previous observations in micellar catalysis and might be a result of the different interplay of headgroup hydration and counterion binding in imidazolium-based surfactants.^43^

**Fig. 4:**
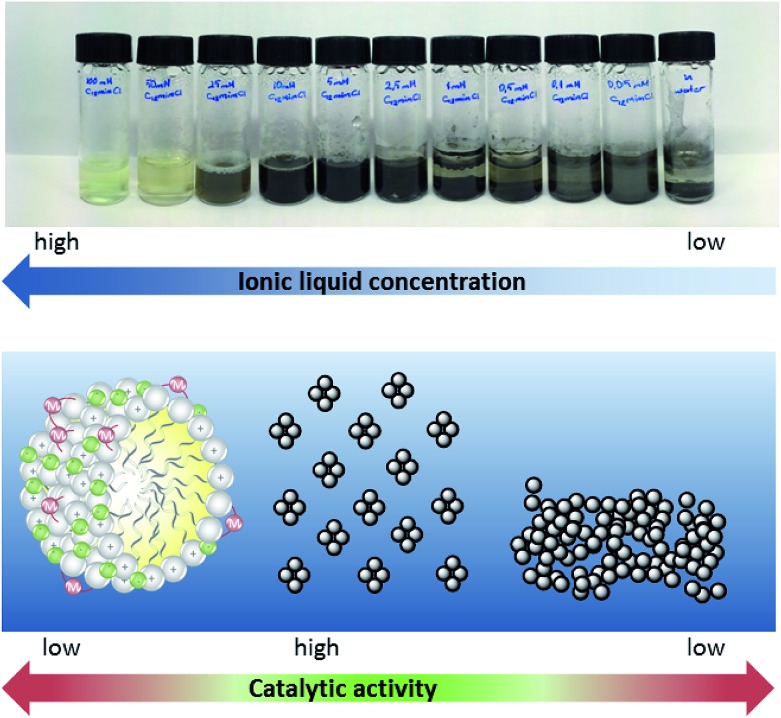
Concentration dependence of palladium-metallomicelles and Pd nanoparticles in aqueous-ionic liquid micellar solution. Conditions: 2 mL [C_12_mimCl] **1** solution, 0.02 mmol Pd_2_(allyl)_2_Cl_2_ and 0.02 mmol K_2_CO_3_; 60 min at 80 °C under air.

As can be easily observed from the appearance of the reaction mixture, this striking concentration effect in the case of surface-active ionic liquid **1** is caused by the presence of different catalytically active palladium species in the aqueous reaction media. At a concentration of 10 mM and below, the black appearance of the reaction mixture indicated the formation of catalytically-active Pd(0) aggregates. This might also explain why the highest yield was obtained with the nitrile-functionalized surface-active ionic liquid: it has been reported that imidazolium-based ionic liquids with CN groups in the side chain can simultaneously act as a solvent and efficiently stabilize palladium nanoparticles, although these observations refer to pure ionic liquids rather than to their aqueous solutions.^44^ At higher concentration a soluble palladium species is present, presumably the NHC complex immobilized on the micellar surface (Fig. 4). This switch between the soluble carbene complex and Pd(0) particles between approx. 10 and 15 mM seems to correlate roughly with the critical micelle concentration (CMC) of the involved ionic liquids that vary between 2 and 13 mM. However, it should be noted that the CMC values are determined for pure aqueous solutions at 25 °C, whereas reactions were run at 80 °C in the presence of salts and reagents, and a certain deviation has to be expected. Additionally, the formed Pd(0) aggregates seem well stabilized in a 10 mM [C_12_mim]Cl **1** solution; however, when the concentration of surfactant further decreases the surfactant tends to agglomerate and form catalytically inactive bulk palladium; hence the yield of ethyl cinnamate gradually decreases below 5 mM.

The consecutive evaluation of reaction conditions showed that a switch to organic bases, *e.g.* diisopropylethylamine (DIPEA) could further improve conversion towards the Heck product, while the extraordinary concentration dependence of [C_12_mim]Cl **1** was retained (see ESI Fig. S1†). A further increase in reaction time to 6 h gave complete conversion of iodobenzene. Under the optimized conditions we were able to isolate ethyl cinnamate in 92% yield using a 10 mM solution of [C_12_mim]Cl **1** in water (Table 2). This provides an efficient protocol for aqueous Heck reaction under aerobic conditions, using only small amounts of surface-active ionic liquid in water without further ligands. To complement this, the reaction could also be optimized for the surface-active ionic liquid [C_12_m_2_im]Cl **2**; here a higher ionic liquid concentration of 100 mM under otherwise similar reaction conditions was found as the optimum and ethyl cinnamate could be isolated in 93% yield. To evaluate the potential catalytic activity, we switched from iodobenzene to bromobenzene and chlorobenzene; however, yields remained low for bromobenzene whereas no conversion was observed for chlorobenzene independent of the ionic liquid used. Although few highly-active water-soluble palladium complexes have been reported for this purpose, the coupling of aryl halides other than iodobenzene clearly remains a challenge, particularly when using ligand-free systems. Moreover, the reduction of the palladium source to lower amounts < 1 mol% resulted in drastically lower yields, thereby clearly showing the limitations of this approach.

**Table 2:** Screening of reaction conditions for surface-active ionic liquids

Entry^*a*^	Ionic liquid	Conc. (mM)	Base	Time (min)	Yield^*b*^ ^,^^*c*^ (%)
1	[C_12_mim]Cl **1**	10	K_2_CO_3_	180	52
2	[C_12_mim]Cl **1**	10	Na_2_CO_3_	180	46
3	[C_12_mim]Cl **1**	10	NaOH	180	11
4	[C_12_mim]Cl **1**	10	NaOAc	180	48
5	[C_12_mim]Cl **1**	10	Et_3_N	180	71
6	[C_12_mim]Cl **1**	10	DIPEA	180	90
7	[C_12_mim]Cl **1**	10	DIPEA	360	>99 (92)
8	[C_12_m_2_im]Cl **2**	100	DIPEA	360	99 (93)
9	Pure H_2_O	—	DIPEA	360	1

^*a*^Reactions were carried out with 1 mmol ArI, 1.5 mmol ethyl acrylate and 1.5 mmol base in 5 mL of aqueous ionic liquid solution at 80 °C under air.

^*b*^Yield determined *via* HPLC using phenol as internal standard.

^*c*^Isolated yield after column chromatography.

Further investigations towards the formation of the catalytically active species were performed in solutions of [C_12_mim]Cl **1** in the presence of K_2_CO_3_ and Pd_2_(allyl)_2_Cl_2_ without the addition of the starting materials. A rapid and temperature-induced formation of Pd(0) was again observed for ionic liquid concentrations below 25 mM when the sample was heated for 60 min at 80 °C under aerobic conditions. At higher concentrations, a soluble palladium species was present (Fig. 4). This was also supported *via* UV-vis spectroscopy, as a disappearance of the band at 305 nm was observed within 60 min at 80 °C for a 5 mM solution, indicating the formation of palladium nanoparticles.^45^ In contrast, no change of the spectrum was observed for a 50 mM solution. ^13^C NMR spectroscopy in D_2_O gave clear evidence for the presence of a NHC–carbene complex in [C_12_mim]Cl **1** at high concentrations, as the typical signal of C-2 emerged at 177 ppm (see ESI Fig. S2†). This concentration-dependent change between the palladium species is different to other literature protocols showing the formation of palladium nanoparticles in aqueous solutions of surface-active ionic liquids. For example Zhu *et al.* reported the formation of well-dispersed palladium colloids in amphiphilic imidazolium-based ionic liquids that acted simultaneously as stabilizers for the nanoparticles and as surfactants generating an emulsion for the hydrophobic substrates.^46^ Palladium nanoparticles were prepared in aqueous solution *via* hydrogenation, and are formed independent of the concentration range of the ionic liquid. When assessing the impact of ionic liquid concentration on the catalytic activity, the authors observed a constant increase in turn-over frequency (TOF) that continued distinctly above the CMC of the respective ionic liquid, and only decreases slowly at very high concentrations.

**Fig. 5:**
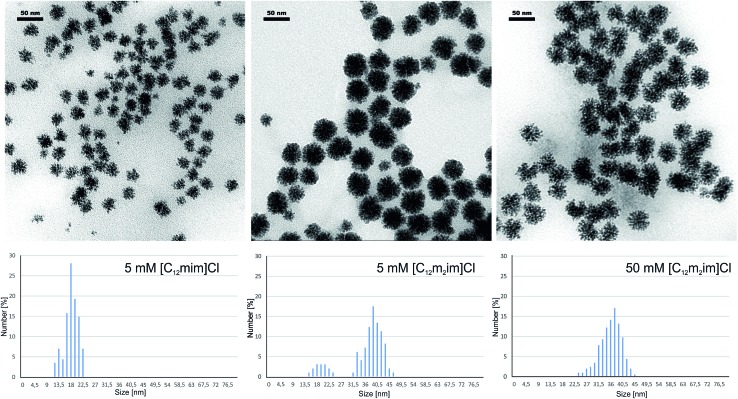
Transmission electron microscopy (TEM) analysis of Pd aggregates and their corresponding size distribution. Left: 5 mM [C_12_mim]Cl **1**, middle: 5 mM [C_12_m_2_im]Cl **2**, right: 50 mM [C_12_m_2_im]Cl **2**; conditions: 2 mL ionic liquid solution, 0.02 mmol Pd_2_(allyl)_2_Cl_2_ and 0.02 mmol K_2_CO_3_; 60 min at 80 °C under air.

The switch between the soluble palladium carbene complex and Pd(0) was further confirmed *via* transmission electron microscopy (TEM) (Fig. 5). In the case of ionic liquid [C_12_mim]Cl **1**, small, spherical Pd(0) nanoparticles are formed at ionic liquid concentrations < 25 mM that aggregate into well-dispersed star-shaped clusters of approx. 20 nm diameter. The unique concentration dependence of the soluble palladium complex and nanoparticles was not found for the C-2 methylated ionic liquid [C_12_m_2_im]Cl **2**. We observed the formation of Pd(0) aggregates over the entire concentration range; however, when comparing the size distribution in a 5 mM [C_12_m_2_im]Cl **2** solution, larger aggregates with approx. 40 nm diameter were formed.

## Conclusion

We presented the application of surface-active imidazolium-based ionic liquids in water as reaction media for Pd-catalyzed cross coupling and found a surprising concentration effect. Our results indicate that the ionic liquid concentration is the key factor affecting the morphology and chemical state of the palladium species and thus for controlling the catalytic activity in Heck-coupling. While an N-heterocyclic carbene complex is formed at higher concentrations, these metallomicelles rapidly decompose into catalytically active Pd(0) nanoclusters at lower surfactant concentrations. This unique behaviour was only observed for amphiphilic ionic liquids that are able to form a carbene species in water and differs significantly from dimethylimidazolium-based ionic liquids or from conventional surfactants. We envision that imidazolium based surfactants can therefore simultaneously act as templates for nanoparticle formation *via* NHC carbene complex formation, but also as stabilizers and surfactants for a consecutive reaction, and more investigations in this regard are currently ongoing in our lab.

## Experimental

### General

Commercially available reagents and solvents were used as received from Sigma Aldrich unless otherwise specified. *N*-Methylimidazole was distilled before use. All ionic liquids were dried for at least 48 h at room temperature and 0.01 mbar before use and were stored under an argon atmosphere. Doubly-distilled deionized water was obtained from a Millipore Milli-Q water purification system (Millipore, USA).

^1^H and ^13^C NMR spectra were recorded on a Bruker Advance UltraShield 400 (400 MHz) spectrometer, using the solvent peak as reference. Elemental analysis was performed at the University of Vienna, Department of Physicochemistry Laboratory for Microanalysis, Währingerstrasse 42, A-1090 Vienna. The FT-IR spectrometer was equipped with a specac MK II Golden Gate Single Reflection ATR unit. TEM studies were performed on a FEI Tecnai G2 20 transmission electron microscope operating at 160 kV. A drop of the dispersion was added on a 300-mesh carbon-coated copper grid and the solvent was subsequently evaporated in air.

HPLC analysis was performed on a Jasco HPLC unit equipped with a PDA detector under reverse-phase conditions. A Maisch ReproSil 100 C18 250 × 4.6, 5 μm was used with MeOH : H_2_O (70 : 30; 0.1% trifluoroethanoic acid) as solvent and a flow of 0.8 cm^3^ min^–1^; detection was at 210 nm, at 30 °C column oven temperature. Calibration curves were prepared in the range from 2.0–0.01 mg mL^–1^ for starting materials and products using phenol as internal standard.

For the determination of the critical micelle concentrations (CMCs) of the surface-active ionic liquids a similar protocol to our previous work was followed.^36^ Solutions at various concentrations of the ionic liquids were prepared with doubly-distilled Millipore Milli-Q water and left under shaking at 360 min^–1^ for 24 h at room temperature (RT) to equilibrate. For conductivity measurements, samples were previously equilibrated at 25 ± 0.1 °C in a HAAKE K15 thermostat. Conductivity measurements were performed on a Mettler Toledo Seven Excellence system, equipped with an InLAB 741-ISM electrode (cell constant *κ* = 0.105). The conductometer was calibrated with a standard KCl solution and two independent measurements were performed. The CMC was calculated as the intersection point of the two linear regimes in the conductivity/concentration graph. Surface tension was determined with the Du Noüy ring method on a Krüss manual tensiometer K6 at room temperature. Each measurement was repeated 5 times.

### Synthesis of ionic liquids

Unfunctionalized imidazolium salts [C_12_mim]Cl **1** and [C_12_m_2_im]Cl **2** were synthesized according to standard methodologies, which include the alkylation of *N*-methylimidazole or dimethylimidazole with the appropriate dodecyl chloride to afford the corresponding imidazolium halide. The imidazolium salts were repeatedly crystallized in order to obtain colorless solids. All ionic liquids were dried for at least 48 h at room temperature and 0.01 mbar before use and were stored under an argon atmosphere.

#### 1-Dodecyl-3-methylimidazolium chloride (**1**)

After following the general procedure the product was repeatedly recrystallized from THF and obtained in 80% yield. ^1^H NMR (400 MHz; CDCl_3_; 22 °C): *δ* (ppm) = 10.57 (1H, s, H(2)-im), 7.54 (1H, s, H(5)-im), 7.35 (1H, s, H(4)-im), 4.27 (2H, t, NCH_2_, *J* = 14.83 Hz), 4.09 (3H, s, NCH_3_), 1.86 (2H, m, NCH_2_*CH*_2_), 1.20 (18H, m, *C*_9_*H*_18_CH_3_), 0.83 (3H, t, NC_11_H_22_*CH*_3_, *J* = 6.36 Hz). ^13^C NMR (100 MHz; CDCl_3_; 22 °C): *δ* (ppm) = 137.35 (d, C(2)-im), 123.76 (d, C(5)-im), 121.83 (d, C(4)-im), 49.83 (t, NCH_2_), 36.38 (q, NCH_3_), 26.09–31.72 (t, C_8_H_16_CH_2_CH_3_), 22.48 (t, NCH_2_CH_2_), 13.93 (q, CH_3_). The data was in accordance with the literature.^33^

#### 1-Dodecyl-2,3-dimethylimidazolium chloride (**2**)

After following the general procedure the product was repeatedly recrystallized from THF and obtained in 92% yield. ^1^H NMR (400 MHz; CDCl_3_; 22 °C): *δ* (ppm) = 7.78 (1H, d, H(5)-im, *J* = 2.08 Hz), 7.44 (1H, d, H(4)-im, *J* = 2.04 Hz), 4.10 (2H, t, NCH_2_, *J* = 14.88 Hz), 3.93 (3H, s, NCH_3_), 2.70 (3H, s, –CCH_3_), 1.67 (2H, m, NCH_2_*CH*_2_), 1.11 (18H, m, CH_2_*C*_9_*H*_18_CH_3_), 0.74 (3H, t, NC_11_H_22_*CH*_3_, *J* = 6.86 Hz). ^13^C NMR (100 MHz; CDCl_3_; 22 °C): *δ* (ppm) = 143.44 (d, C(2)-im), 123.31 (d, C(5)-im), 121.15 (d, C(4)-im), 49.84 (t, NCH_2_), 35.88 (q, NCH_3_), 31.76–26.42 (t, C_8_H_16_CH_2_CH_3_), 22.55 (t, NCH_2_CH_2_), 14.00 (q, CH_3_), 10.47 (q, CH_3_). The data was in accordance with the literature.^33^

Functionalized imidazolium salts **3–8** were synthesized through a two step procedure involving the alkylation of 1-dodecylimidazole which was previously synthesized following a procedure reported in the literature and distilled before use with the appropriate halide.^47^

#### 1-Dodecyl-3-(2-hydroxyethyl)-imidazolium chloride (**3**)

*N*-Dodecylimidazole (5.00 g, 21.2 mmol) was mixed with 2-chloroethanol (2.90 g, 36.0 mmol). The mixture was heated to 50 °C for 76 h. After evaporation of the remaining volatile materials, the crude product was twice crystallized from anhydrous THF to yield 1-dodecyl-2-(2-hydroxyethyl)-imidazolium chloride (**3**) as a colourless solid in 74% yield. ^1^H NMR (400 MHz, CDCl_3_; 22 °C): *δ* (ppm) = 9.72 (1H, s, H(2)-im), 7.68 (1H, s, H(5)-im), 7.32 (1H, s, H(4)-im), 5.13 (1H, m, OH), 4.53 (2H, t, HOCH_2_*CH*_2_N, *J* = 4.31 Hz), 4.41 (2H, t, NCH_2_, *J* = 7.21 Hz), 3.83 (2H, t, HO*CH*_2_CH_2_N, *J* = 4.13 Hz), 1.77 (2H, m, NCH_2_*CH*_2_), 1.13 (18H, m, *C*_9_*H*_18_CH_3_), 0.75 (3H, t, NC_11_H_22_*CH*_3_, *J* = 7.13 Hz). ^13^C NMR (100 MHz; CDCl_3_; 22 °C): *δ* (ppm) = 138.10 (d, C(2)-im), 124.12 (d, C(5)-im), 121.72 (d, C(4)-im), 62.69 (t, HO*CH*_2_CH_2_N), 52.00 (t, HOCH_2_*CH*_2_N), 50.13 (t, NCH_2_), 30.11–26.74 (t, C_8_H_16_CH_2_CH_3_), 22.48 (t, NCH_2_*CH*_2_), 13.93 (q, CH_3_). The data was in accordance with the literature.^40^

#### 1-Dodecyl-3-(2-methoxyethyl)-imidazolium chloride (**4**)

*N*-Dodecylimidazole (5.00 g, 21.2 mmol) was mixed with 1-chloro-2-methoxyethane (3.60 g, 38.1 mmol). The mixture was heated to 80 °C for 76 h. After evaporation of the remaining volatile materials, the crude product was twice crystallized from anhydrous THF to yield 1-dodecyl-3-(2-methoxyethyl)-imidazolium chloride (**4**) as a colourless solid in 78% yield. ^1^H NMR (400 MHz, CDCl_3_; 22 °C): *δ* (ppm) = 10.67 (1H, s, H(2)-im), 7.49 (1H, s, H(5)-im), 7.24 (1H, s, H(4)-im), 4.63 (2H, t, CH_3_OCH_2_*CH*_2_*N*, *J* = 4.66 Hz), 4.27 (2H, t, NCH_2_, *J* = 7.45 Hz), 3.75 (2H, t, CH_3_O*CH*_2_CH_2_N, *J* = 4.65 Hz), 3.35 (3H, s, OCH_3_), 1.88 (2H, m, NCH_2_*CH*_2_), 1.22 (18H, m, *C*_9_*H*_18_CH_3_), 0.84 (3H, t, NC_11_H_22_*CH*_3_, *J* = 7.21 Hz). ^13^C NMR (100 MHz; CDCl_3_; 22 °C): *δ* (ppm) = 136.97 (d, C(2)-im), 123.23 (d, C(5)-im), 121.58 (d, C(4)-im), 70.36 (t, CH_3_O*C*_2_CH_2_N), 58.85 (q, OCH_3_), 49.49 (t, NCH_2_), 31.70 (t, CH_3_OCH_2_*CH*_2_N), 30.22–26.34 (t, *C*_8_*H*_16_CH_2_CH_3_), 22.60 (t, NCH_2_*CH*_2_), 13.91 (q, CH_3_). ATR-IR (neat): *λ* = 3394, 2922, 2853, 1563, 1465, 1166, 1120 cm^–1^. C_18_H_35_ClN_2_O: calcd C 65.33, H 10.66, N 8.46; found C 65.36, H 11.16, N 8.34.

#### 1-Dodecyl-3-(2-(methylthio)ethyl)-imidazolium chloride (**5**)

Dodecylimidazole (5.00 g, 21.2 mmol) was mixed with (2-chloroethyl)(methyl)sulfane (2.46 g, 22.2 mmol). The mixture was heated to 80 °C for 76 h. After evaporation of the remaining volatile materials, the crude product was twice crystallized from anhydrous THF to yield 1-dodecyl-3-(2-(methylthio)ethyl)-imidazolium chloride (**5**) as a colourless solid in 89% yield. ^1^H NMR (400 MHz, CDCl_3_; 22 °C): *δ* (ppm) = 10.52 (1H, s, H(2)-im), 7.80 (1H, s, H(5)-im), 7.37 (1H, s, H(4)-im), 4.56 (2H, t, NCH_2_, *J* = 6.63 Hz), 4.18 (2H, t, CH_3_SCH_2_*CH*_2_*N*, *J* = 7.58 Hz), 2.95 (2H, t, CH_3_S*CH*_2_CH_2_N, *J* = 4.7.56 Hz), 2.08 (3H, s, SCH_3_), 1.80 (2H, m, NCH_2_*CH*_2_), 1.12 (18H, m, *C*_9_*H*_18_CH_3_), 0.75 (3H, t, NC_11_H_22_*CH*_3_, *J* = 7.09 Hz). ^13^C NMR (100 MHz; CDCl_3_; 22 °C): *δ* (ppm) = 138.00 (d, C(2)-im), 123.07 (d, C(5)-im), 121.61 (d, C(4)-im), 50.05 (t, CH_3_SCH_2_*CH*_2_N), 48.59 (t, NCH_2_), 31.76 (t, CH_3_S*CH*_2_CH_2_N), 30.44–26.34 (t, *C*_8_*H*_16_CH_2_CH_3_), 22.53 (t, NCH_2_*CH*_2_), 15.51 (q, SCH_3_), 14.05 (q, CH_3_). ATR-IR (neat): *λ* = 3378, 2916, 2847, 1573, 1560, 1467, 1173 cm^–1^. C_18_H_35_ClN_2_S: calcd C 62.30, H 10.17, N 8.07; found C 62.14, H 10.41, N 8.06.

#### 1-Cyanomethyl-3-dodecyl-imidazolium chloride (**6**)

Dodecylimidazole (5.00 g, 21.2 mmol) was mixed with 2-chloroacetinitril (1.59 g, 21.2 mmol). The mixture was heated to 80 °C for 30 min. After evaporation of the remaining volatile materials, the crude product was crystallized from anhydrous THF to yield 1-cyanomethyl-3-dodecyl-imidazolium chloride (**6**) as a colourless solid in 80% yield. ^1^H NMR (400 MHz, CDCl_3_; 22 °C): *δ* (ppm) = 10.55 (1H, s, H(2)-im), 8.25 (1H, s, H(5)-im), 7.44 (1H, s, H(4)-im), 6.26 (s, 2H, N*CH*_2_CN), 4.24 (2H, t, NCH_2_, *J* = 7.50 Hz), 1.85 (2H, m, NCH_2_*CH*_2_), 1.12 (18H, m, *C*_9_*H*_18_CH_3_), 0.85 (3H, t, NC_11_H_22_*CH*_3_, *J* = 6.50 Hz). ^13^C NMR (100 MHz; CDCl_3_; 22 °C): *δ* (ppm) = 135.67 (d, C(2)-im), 121.95 (d, C(5)-im), 120.59 (d, C(4)-im), 112.35 (s, CN), 48.73 (t, NCH_2_), 35.85 (t, N*CH*_2_CN), 30.02–24.43 (t, *C*_8_*H*_16_CH_2_CH_3_), 20.79 (t, NCH_2_*CH*_2_), 12.12 (q, CH_3_). ATR-IR (neat): *λ* = 3114, 3079, 2918, 2852, 2254, 1549, 1151 cm^–1^. C_17_H_30_ClN_3_: calcd C 65.47, H 9.70, N 13.47; found C 65.05, H 9.81, N 13.57.

#### 1-Dodecyl-3-(2-pyridylmethyl)imidazolium chloride (**7**)

2-(Chloromethyl)pyridine hydrochloride (4.92 g, 30 mmol) was dissolved in water (165 mL). Solid NaHCO_3_ was added (5.02 g, 60 mmol) under magnetic stirring, and the mixture was repeatedly extracted with dichloromethane. The combined organic layers were washed with brine, dried over Na_2_SO_4_ and the solvent was removed under reduced pressure. 2-(Chloromethyl)pyridine was obtained as a light brown liquid in 97% yield and directly subjected to the next step. 2-(Chloromethyl)pyridine (3.87 g, 25 mmol) and freshly distilled 1-dodecylimidazol (5.91 g, 25 mmol) were heated with an oil bath at 80 °C in a three-neck round-bottomed flask equipped with a stirrer bar and reflux condenser under a dry argon atmosphere for 48 h until NMR indicated complete conversion. The crude product was repeatedly washed with anhydrous ethyl acetate. Remaining solvent traces were removed under reduced pressure (10^–2^ mbar) to yield 1-dodecyl-3-(2-pyridylmethyl)imidazolium chloride (**7**) as a light brown viscous liquid in 95% yield. ^1^H NMR (400 MHz, CDCl_3_; 22 °C): *δ* (ppm) = 10.68 (1H, s, H(2)-im), 8.40 (1H, s, H(5)-im), 7.78 (1H, s, H(4)-im), 7.70 (1H, s), 7.63 (1H, m), 7.27 (1H, m), 7.21 (1H, m), 5.7 (2H, s, NCH_2_), 4.23 (2H, t, N*CH*_2_CH_2_, *J* = 7.5 Hz), 1.86 (2H, m, NCH_2_*CH*_2_), 1.10 (18H, m, *C*_9_*H*_18_CH_3_), 0.80 (3H, t, NC_11_H_22_*CH*_3_, *J* = 6.36 Hz). ^13^C NMR (100 MHz, CDCl_3_; 22 °C): *δ* (ppm) = 152.09 (s), 149.76 (d, C(2)-im), 137.02 (2d, C(5)-im, C(4)-im), 124.30 (d), 122.74 (2d), 121.46 (d), 54.05 (t, NCH_2_), 50.21 (t, NCH_2_CH_2_), 31.77 (q, NCH_3_), 30.29–26.31 (t, C_8_H_16_CH_2_CH_3_), 22.60 (t, NCH_2_CH_2_), 14.06 (q, CH_3_). C_21_H_34_ClN_3_: calcd C 69.30, H 9.42, N 11.55; C_21_H_34_ClN_3_·0.6H_2_O: calcd C 67.30, H 9.47, N 11.21; found C 67.29, H 9.72, N 11.22.

#### 1-Allyl-3-dodecyl-imidazolium chloride (**8**)

Dodecylimidazole (5.00 g, 21.2 mmol) was mixed with allyl chloride (4.05 g, 52.9 mmol). The mixture was heated to 50 °C for 24 h. After evaporation of the remaining volatile materials, the crude product was crystallized from anhydrous THF to yield 1-allyl-3-dodecyl-imidazolium chloride (**8**) as a colourless solid in 94% yield. ^1^H NMR (400 MHz, CDCl_3_; 22 °C): *δ* (ppm) = 10.75 (1H, s, H(2)-im), 7.45 (1H, s, H(5)-im), 7.42 (1H, s, H(4)-im), 5.69 (m, 1H, allyl-CH), 5.40 (2H, t, NCH_2_, *J* = 7.50 Hz), 5.01 (2H, d, N*CH*_2_CHCH_2_, *J* = 6.60 Hz), 4.28 (m, 2H, allyl-CH_2_), 1.85 (2H, m, NCH_2_*CH*_2_), 1.12 (18H, m, *C*_9_*H*_18_CH_3_), 0.82 (3H, t, NC_11_H_22_*CH*_3_, *J* = 6.60 Hz). ^13^C NMR (100 MHz; CDCl_3_; 22 °C): *δ* (ppm) = 137.10 (d, C(2)-im), 130.08 (d, allyl-CH), 122.31 (d, C(5)-im), 122.15 (d, C(4)-im), 122.10 (t, allyl-CH_2_) 51.71 (t, NCH_2_), 49.94 (t, N*CH*_2_CHCH_2_), 31.69 (t, CH_3_S*CH*_2_CH_2_N), 30.17–26.10 (t, *C*_8_*H*_16_CH_2_CH_3_), 22.47 (t, NCH_2_*CH*_2_), 13.94 (q, CH_3_). ATR-IR (neat): *λ* = 3382, 2922, 2852, 1645, 1560, 1465, 992, 940 cm^–1^. C_18_H_33_ClN_2_: calcd C 69.09, H 10.63, N 8.95; C_18_H_33_ClN_2_·0.65H_2_O: calcd C 66.60, H 10.65, N 8.63; found C 66.68, H 10.49, N 8.30.

#### Application of surface-active ionic liquids in the Heck reaction

The representative procedure for the Heck reaction of ethyl acrylate **9** and iodobenzene **10** in aqueous solution of ionic liquids for Heck cross-coupling is as follows: a screw-cap vial with a Teflon seal was charged with 0.02 mmol Pd_2_(allyl)_2_Cl_2_, 1.5 mmol K_2_CO_3_ and a magnetic stirring bar. Aqueous-ionic liquid micellar solution (5.0 mL) was added and the reaction mixture was stirred at 80 °C for 15 minutes. Freshly distilled ethyl acrylate (1.5 mmol) and iodobenzene (1.0 mmol) were added and the mixture was reacted at 80 °C until completion. The reaction mixture was extracted with light petrol (fraction 40–60 °C) three times. The combined organic phases were once extracted with 2 N HCl and filtered over a batch of silica. The solvent was removed under reduced pressure (40 °C, 50 mbar) to yield ethyl cinnamate in 93% yield. ^1^H NMR (400 MHz, CDCl_3_; 22 °C): *δ* (ppm) = 7.86 (1H, d, *J* = 16.04 Hz), 7.53 (2H, m), 7.38 (3H, m), 6.37 (1H, d, *J* = 16.09 Hz), 4.27 (2H, q, *J* = 7.02 Hz), 1.34 (3H, t, *J* = 7.10 Hz). The data was in accordance with the literature.^48^

## Conflicts of interest

There are no conflicts to declare.

## Supplementary Material

Supplementary information
