# Supplementary material for: Surface-active ionic liquids for palladium-catalysed cross coupling in water: effect of ionic liquid concentration on the catalytically active species
Source: RSC Adv. 2017 Aug 22;7(65):41144–51. doi: 10.1039/c7ra07757b (PMC5708357; doi:10.1039/c7ra07757b)
Supplement: Supplementary file 1 [file RA-007-C7RA07757B-s001.pdf]

Electronic Supplementary Material (ESI) for RSC Advances.
This journal is © The Royal Society of Chemistry 2017

Electronic supporting information

Surface-active ionic liquids for Palladium-catalysed cross coupling in water: Effect of ionic liquid concentration on the catalytically active species

Meltem Taskin,[a] Alice Cognigni,[a] Ronald Zirbs,[b] Erik Reimhult[b] and Katharina Bica[a,*]

[a] Institute of Applied Synthetic Chemistry, Vienna University of Technology, Getreidemarkt 9/163, 1060 Vienna (Austria), Fax: +43 1 58801 16360; Tel: +43 1 58801 163601. E-mail: katharina.bica@tuwien.ac.at or katharina.schroeder@tuwien.ac.at .

[b] Group for Biologically Inspired Materials, Institute of Nanobiotechnology (DNBT), University of Natural Resources and Life Sciences, Muthgasse 11, 1190 Vienna, Austria.

1. Table S1: Critical micelle concentrations for all involved ionic liquids 2

2. Figure S1: Concentration dependence of the Heck reaction of ethyl acrylate 9 and iodobenzene 10 in aqueous solution of ionic liquids [C_{12}mim]Cl 1 using DBU as base... 3

3. Figure S2: Copy of ^{13}C NMR spectroscopy showing the formation of a Pd-carbene at 177 ppm......... 4

1. Table S1: Critical micelle concentrations for all involved ionic liquids

	CMC [mM][a]	
**Ionic Liquid**	**Conductivity[b]**	**Surface tension[c]**
**1**	14.53 [1]	13.25 [1]
**2**	13.75 [2]	10.33 [2]
**3**	2.69 [3]	2.31 [3]
**4**	4.32	3.24
**5**	5.52	4.33
**6**	5.69	5.07
**7**	5.04	4.91
**8**	4.17	5.61

[a] Solutions were prepared with doubly-distilled Millipore Milli-Q water. Samples were equilibrated at 25 °C with a HAAKE K15 thermostat before measurements. [b] Conductivity measurement were performed on a Mettler Toledo SevenExcellence system. InLAB® 741-ISM electrode (cell constant k = 0.105). The conductimeter was calibrated with a standard KCl solution and measurements were performed in duplicate; [c] Surface tension was determined with the Du Noüy ring method on a Krüss tensiometer at RT. Each measurement was repeated 5 times.

[1] A. Cognigni, P. Gaertner, R. Zirbs, H. Peterlik, K. Prochazka, C. Schröder and K. Bica, *Phys. Chem. Chem. Phys.*, 2016, **18**, 13375–13384.

[2] A. Cognigni, S. Kampichler and K. Bica, *J. Colloid Interface Sci.*, 2017, **492**, 136–145.

[3] X.-f. Liu, L.-l. Dong and Y. Fang, *J. Surfactants Deterg.*, 2011, **14**, 203–210.

2. **Figure S1: Concentration dependence of the Heck reaction of ethyl acrylate 9 and iodobenzene 10 in aqueous solution of ionic liquids [C_{12}mim]Cl 1 using DBU as base.**

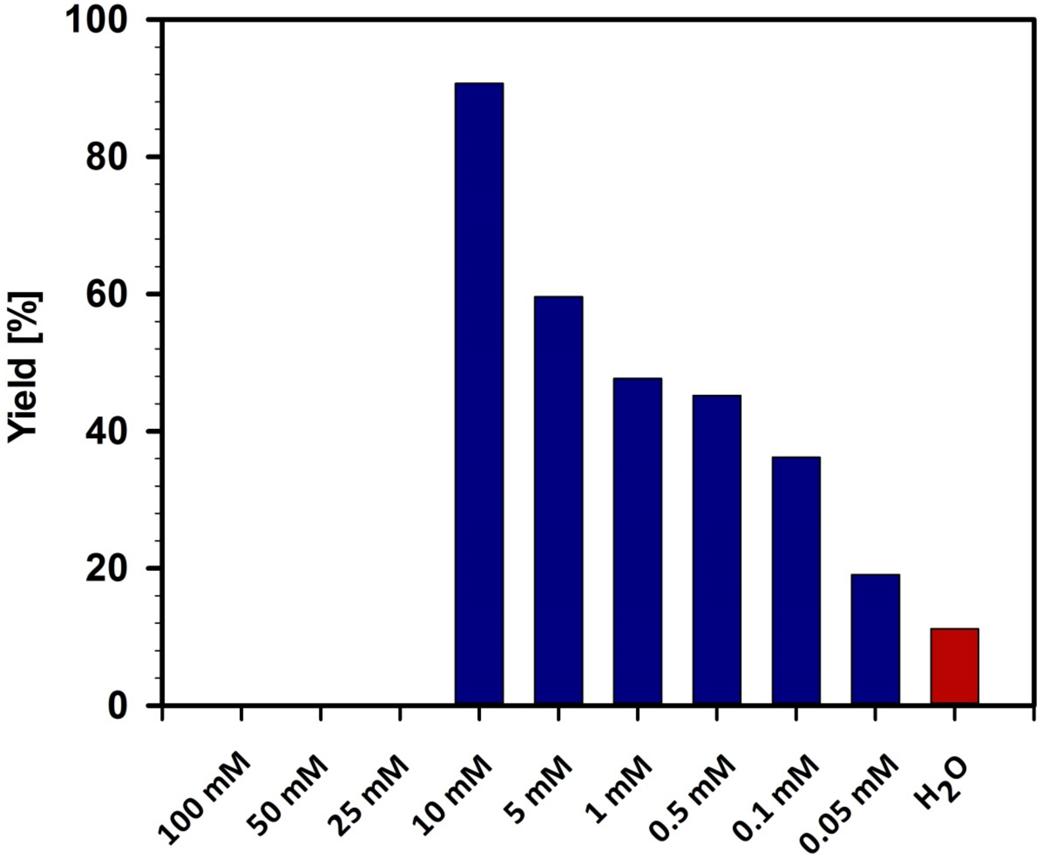

3. **Figure S2: Copy of ^{13}C NMR spectroscopy showing the formation of a Pd-carbene at 177 ppm.**

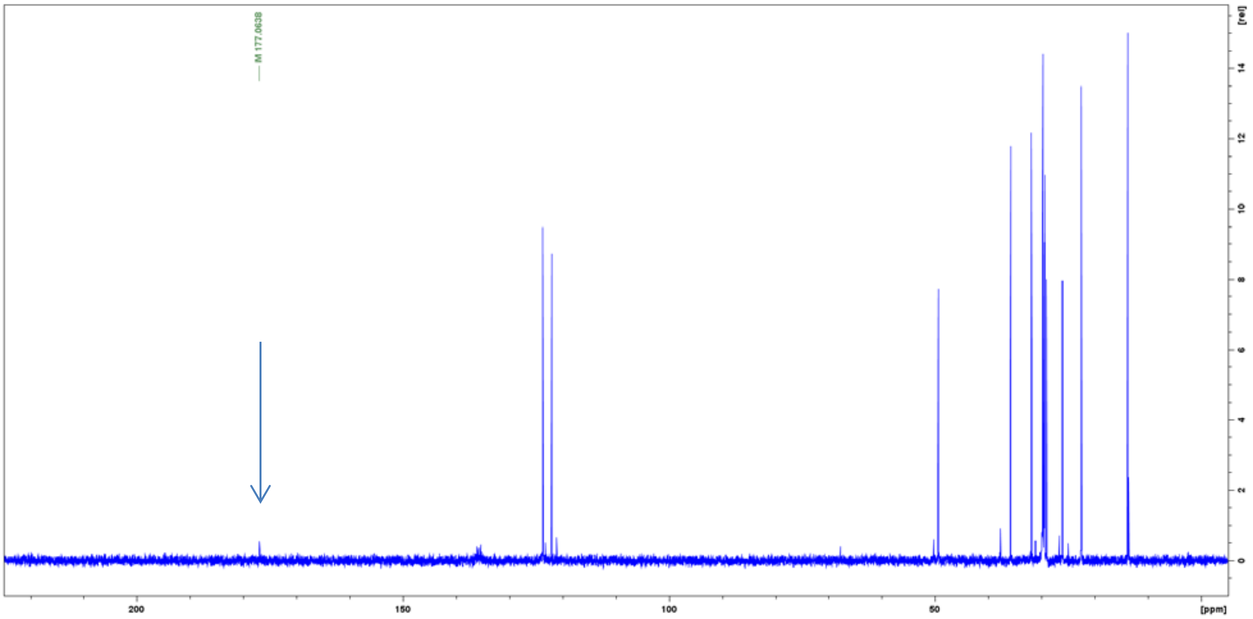

Conditions: 2 mL [C_{12}mimCl] **2** solution (50 mM) in D_2O, 0.02 mmol $Pd_2allyl_2Cl_2$ and 0.02 mmol K_2CO_3; 30 min at 80 °C under air.

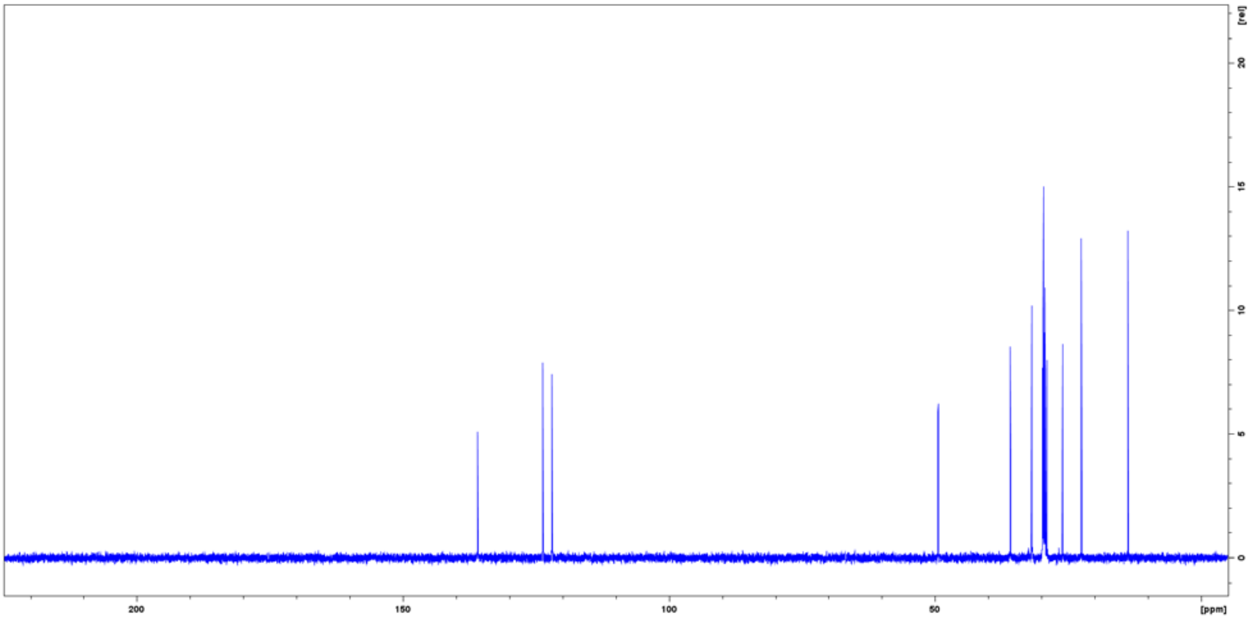

Conditions: [C_{12}mimCl] **2** solution (50 mM) in D_2O
